# Exercise Biomechanics and Physiology

**DOI:** 10.3390/life11020159

**Published:** 2021-02-19

**Authors:** Jose I. Priego-Quesada

**Affiliations:** 1 Research Group in Sports Biomechanics (GIBD), Department of Physical Education and Sports, University of Valencia, 46010 Valencia, Spain; j.ignacio.priego@uv.es; 2 Biophysics and Medical Physics Group, Department of Physiology, University of Valencia, 46010 Valencia, Spain

Biomechanics was defined by Hatze in 1974 as the study of the movement of living things using the science of mechanics [1]. However, a definition that may more fully explain what the research community understand by biomechanics is that proposed by the European Society of Biomechanics: “the study of forces acting on and generated within a body and the effects of these forces on the tissues, fluid or materials used for the diagnosis, treatment or research purposes” [2]. Furthermore, human physiology has the objective to explain the physical and chemical factors of the human body that make it a living being [3]. Currently, from the sport science perspective, most studies combine both disciplines to answer research questions in a more holistic way. In other cases, one or other of the disciplines tries to estimate a parameter using alternative techniques for reasons of accessibility or ease of use.

A large number of recent studies could be referred to as examples of how the combination of both disciplines results in a more complete approach to explaining movement, its effects and its consequences. For example, a very current and controversial topic is the effect of running footwear on long-distance runners’ performance. Here, various studies have assessed different shoe models exploring both kinds of variables, the physiological (e.g., oxygen consumption, running economy) and the biomechanical parameters (e.g., stride length, plantar flexion velocity, and center of mass vertical oscillation) [4,5]. Another example is in cycling, where muscle activation, measured using electromyography and kinematics, are combined to better understand the effect of factors such as posture, components or exercise intensity [6,7,8,9]. A good example of the interaction between both disciplines is present in this Special Issue (“Exercise Biomechanics and Physiology”, *Life*). Wannop and colleagues [10] explored the effect of surface stiffness of artificial turf systems on athlete performance. They assessed both the physiological (oxygen consumption and running economy) and biomechanical parameters (kinematic variables). This approach allows the authors to observe how the type of surface affects different variables related with performance in different movements (running, sprint, vertical jump, and agility with movement changes [10]).

There are also a number of studies that have tried to estimate some physiological parameters with biomechanical measures or vice versa. An example of this are the studies carried out with infrared thermography where, due to its ease of use, the aim is to estimate plantar pressure, asymmetries in the performance of forces or sports technique [11,12,13,14]. Another example is the study published in this Special Issue by Mendonça Teixeira and colleagues [15]. Due to the expensive and time-consuming characteristics of isokinetic dynamometry for screening injury risk, they analyzed the association between muscular strength imbalances and skin temperature [15]. In this case, however, they concluded that the skin temperature differences between hamstrings and quadriceps could be more related to thermoregulatory factors than strength imbalances [15].

Clearly there are also studies in which, due to the objectives or specialism of the authors, focus on the parameters of a single discipline. This Special Issue reflects this, with some studies focused only on physiological parameters [16,17,18,19], and others on biomechanical parameters [20,21,22,23,24]. However, being aware that multidisciplinarity enriches scientific work [25], we have sought to emphasize this holistic approach in this special issue.

## References

[1] Hatze H. (1974). The Meaning of the Term “Biomechanics”. J. Biomech..

[2] Hamill J. (2007). Biomechanics Curriculum: Its Content and Relevance to Movement Sciences. Quest.

[3] Guyton A.C., Hall J.E. (2006). Textbook of Medical Physiology.

[4] Hunter I., McLeod A., Valentine D., Low T., Ward J., Hager R. (2019). Running Economy, Mechanics, and Marathon Racing Shoes. J. Sports Sci..

[5] Cigoja S., Fletcher J.R., Esposito M., Stefanyshyn D.J., Nigg B.M. (2021). Increasing the Midsole Bending Stiffness of Shoes Alters Gastrocnemius Medialis Muscle Function during Running. Sci. Rep..

[6] Bourdon E., Mavor M., Hay D.C. (2017). Assessment of Three-Dimensional Trunk Kinematics and Muscle Activation during Cycling with Independent Cranks. J. Sports Sci. Med..

[7] Holliday W., Theo R., Fisher J., Swart J. (2019). Cycling: Joint Kinematics and Muscle Activity during Differing Intensities. Sports Biomech..

[8] Brand A., Sepp T., Klöpfer-Krämer I., Müßig J.A., Kröger I., Wackerle H., Augat P. (2020). Upper Body Posture and Muscle Activation in Recreational Cyclists: Immediate Effects of Variable Cycling Setups. Res. Q. Exerc. Sport.

[9] Millour G., Duc S., Puel F., Bertucci W. (2020). Physiological, Biomechanical, and Subjective Effects of Medio-Lateral Distance between the Feet during Pedalling for Cyclists of Different Morphologies. J. Sports Sci..

[10] Wannop J., Kowalchuk S., Esposito M., Stefanyshyn D. (2020). Influence of Artificial Turf Surface Stiffness on Athlete Performance. Life.

[11] Gil-Calvo M., Jimenez-Perez I., Priego Quesada J.I., Lucas-Cuevas A.G., Llana-Belloch S., Pérez-Soriano P. Could Skin Temperature Predict Ankle Eversion after Running?. Proceedings of the Book of Abstracts of the 21st annual Congress of the European College of Sport Science.

[12] Gil-Calvo M., Herrero-Marco J., de González-Peña R.J., Perez-Soriano P., Priego-Quesada J.I. (2020). Acute Effect of Induced Asymmetrical Running Technique on Foot Skin Temperature. J. Therm. Biol..

[13] Trecroci A., Formenti D., Ludwig N., Gargano M., Bosio A., Rampinini E., Alberti G. (2018). Bilateral Asymmetry of Skin Temperature Is Not Related to Bilateral Asymmetry of Crank Torque during an Incremental Cycling Exercise to Exhaustion. PeerJ.

[14] Sanchis-Sanchis R., Priego-Quesada J.I., Ribas-Garcia V., Carpes F.P., Encarnacion-Martinez A., Perez-Soriano P. (2020). Effects of Asymmetrical Exercise Demands on the Symmetry of Skin Temperature in Archers. Physiol. Meas..

[15] Teixeira R.M., Dellagrana R.A., Priego-Quesada J.I., Machado J.C.B., Da Silva J.F., Dos Reis T.M.P., Rossato M. (2020). Muscular Strength Imbalances Are not Associated with Skin Temperature Asymmetries in Soccer Players. Life.

[16] Balatskyi V.V., Palchevska O.L., Bortnichuk L., Gan A.-M., Myronova A., Macewicz L.L., Navrulin V.O., Tumanovska L.V., Olichwier A., Dobrzyn P. (2020). β-Catenin Regulates Cardiac Energy Metabolism in Sedentary and Trained Mice. Life.

[17] Lu W.-A., Chen Y.-S., Wang C.-H., Kuo C.-D. (2020). Effect of a Single Session of Tai Chi Chuan Practice on Glucose and Lipid Metabolism and Related Hormones. Life.

[18] Ruiz-Moreno C., Lara B., Gutiérrez-Hellín J., González-García J., Del Coso J. (2020). Time Course and Magnitude of Tolerance to the Ergogenic Effect of Caffeine on the Second Ventilatory Threshold. Life.

[19] Ramamoorthi R., Gahreman D., Skinner T., Moss S. (2021). Development of Sham Yoga Poses to Assess the Benefits of Yoga in Future Randomized Controlled Trial Studies. Life.

[20] Borges A.L.S., Piva A.M.D.O.D., Concílio L.R.D.S., Paes-Junior T.J.D.A., Tribst J.P.M. (2020). Mouthguard Use Effect on the Biomechanical Response of an Ankylosed Maxillary Central Incisor during a Traumatic Impact: A 3-Dimensional Finite Element Analysis. Life.

[21] Deng L., Zhang X., Xiao S., Yang Y., Li L., Fu W. (2020). Changes in the Plantar Flexion Torque of the Ankle and in the Morphological Characteristics and Mechanical Properties of the Achilles Tendon after 12-Week Gait Retraining. Life.

[22] Duca M., Trecroci A., Perri E., Formenti D., Alberti G. (2020). Kinematics and Kinetics of Bulgarian-Bag-Overloaded Sprints in Young Athletes. Life.

[23] Aguirre-Betolaza A.M., Mujika I., Loprinzi P., Corres P., Gorostegi-Anduaga I., Maldonado-Martín S. (2020). Physical Activity, Sedentary Behavior, and Sleep Quality in Adults with Primary Hypertension and Obesity before and after an Aerobic Exercise Program: EXERDIET-HTA Study. Life.

[24] Yun H., Lee J.-H., Choi I.-R. (2020). Effects of Kinesiology Taping on Shoulder Posture and Peak Torque in Junior Baseball Players with Rounded Shoulder Posture: A Pilot Study. Life.

[25] Piggott B., Müller S., Chivers P., Papaluca C., Hoyne G. (2019). Is Sports Science Answering the Call for Interdisciplinary Research? A Systematic Review. Eur. J. Sport Sci..

